# *Gazella arabica dareshurii*: a remarkable relict population on Farur Island, Iran

**DOI:** 10.1186/s12862-021-01943-1

**Published:** 2021-11-28

**Authors:** Davoud Fadakar, Mojdeh Raam, Hannes Lerp, Ali Ostovar, Hamid Reza Rezaei, Eva V. Bärmann

**Affiliations:** 1 Department of Natural Resources, Isfahan University of Technology, Isfahan, Iran; 2 Hormozgan Department of Environment, Hormozgan, Iran; 3 Natural History Collections, Museum Wiesbaden, Friedrich-Ebert-Allee 2, 65185 Wiesbaden, Germany; 4 Department of Fishery and Environmental Science, Gorgan University of Agricultural Sciences and Natural Resources, Gorgan, Iran; 5 Zoological Research Museum Alexander Koenig, Adenauerallee 160, 53113 Bonn, Germany

**Keywords:** Ungulate, Mountain gazelles, Vicariance, Last glacial maximum, Persian Gulf

## Abstract

**Background:**

The islands in the Persian Gulf are home to several species of gazelles, i.e., *Gazella bennettii*, *G. subgutturosa*, and a new subspecies of Mountain gazelles which was discovered on Farur Island and described for the first time in 1993 as *Gazella gazella dareshurii*. Later, phylogenetic analyses showed that the Mountain gazelles consist of two species: *G. gazella* and *G. arabica*. As the Farur gazelles are more closely related to the Arabian forms of the Mountain gazelles, this subspecies is regarded to be *G. arabica dareshurii*. Until now, the origin of this subspecies has been an enigma.

**Results:**

Here, we used mitochondrial cyt *b*, two nuclear introns (CHD2 and ZNF618), and morphological data to address this question by investigating the taxonomic position of the Farur gazelles. The results show that this population is monophyletic and split from other *G. arabica* populations probably 10,000 BP.

**Conclusions:**

It is a natural relict population that was trapped on the island due to the rising sea levels of the Persian Gulf after the Last Glacial Maximum. Intermittent drought and flooding are suggested to be the main factors balancing population growth in the absence of natural predators on this monsoon-influenced island. Conservation actions should focus on preserving the natural situation of the island (cease introducing mesquite tree and other invasive species, stop building new construction and roads, and caution in providing water sources and forage), and possibly introducing individuals to other islands (not inhabited by gazelles) or to fenced areas on the Iranian mainland (strictly isolated from other gazelle populations) when the population reaches the carrying capacity of the island.

## Background

**Fig. 1:**
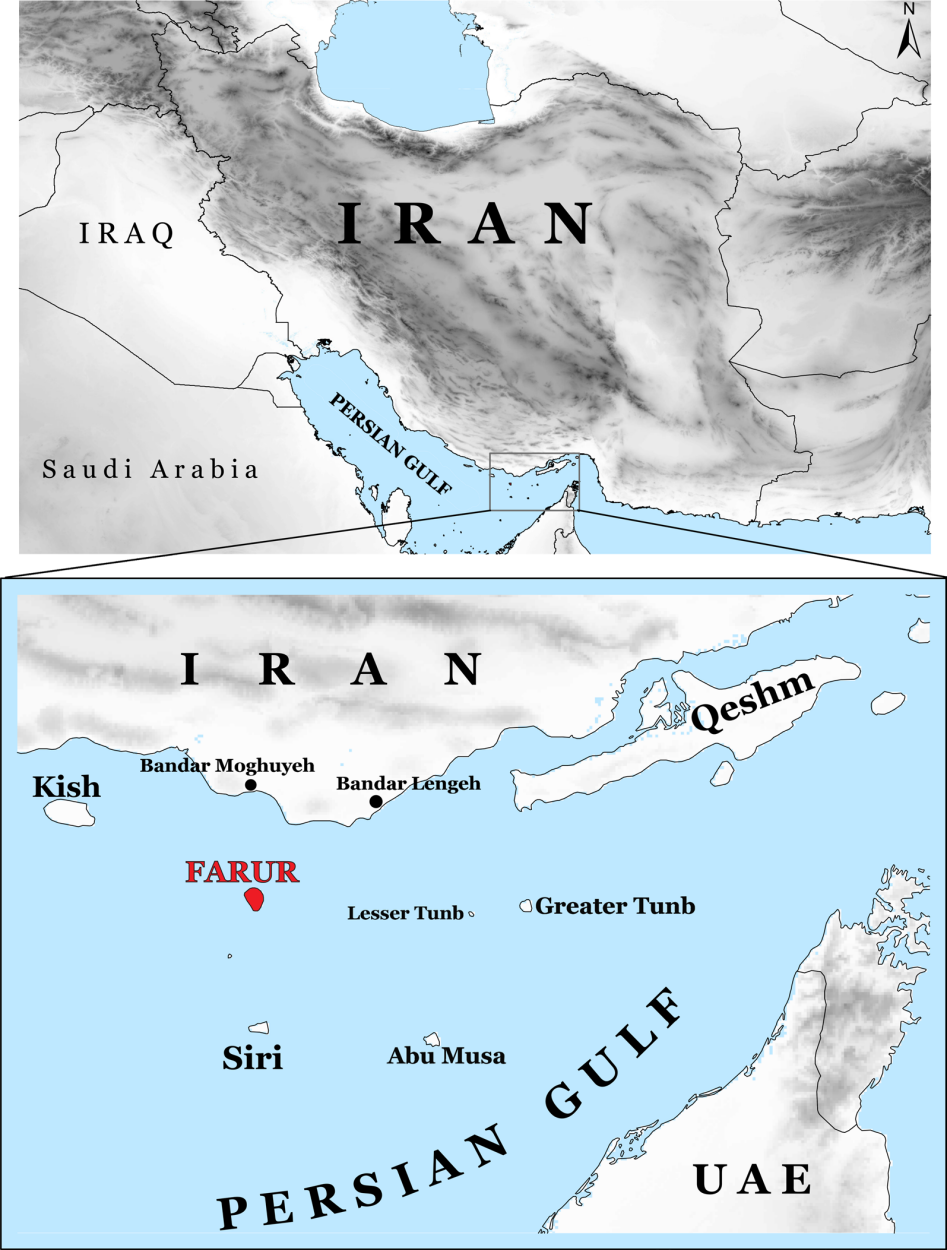
Location of Farur Island in the Persian Gulf. The background hillshade was made using the Shuttle Radar Topography Mission (SRTM) elevation model (http://srtm.csi.cgiar.org) in QGIS version 3.10; country boundaries were downloaded from DIVA-GIS dataset (http://www.diva-gis.org/Data)

After the last glacial maximum (LGM), islands in the Persian Gulf emerged due to the sea-level change at about 18,000 BP, which led to the gradual flooding of the dry gulf basin [1, 2]. Several gazelle species exist on those isolated islands (Fig. 1), including jebeer (*Gazella bennettii*) on Qeshm, Hengam, Hormoz, Larak, and Lavan islands [3], goitered or Persian gazelle (*G. subgutturosa*) on Siri, Kharg, and introduced from Kharg to Kish Island [3–5], and Mountain gazelle or idmi on the small island of Farur [6, 7].

*G. bennettii* and *G. subgutturosa* also inhabit the Iranian mainland [3–5, 8–12], but Mountain gazelles in Iran only exist on Farur Island. The geographically closest populations of Arabian mountain gazelles inhabit the Arabian mainland south of the Persian Gulf [13]. Farur gazelles were unknown to science until 1993, when they were described as a distinct subspecies (*dareshurii*) of mountain gazelles by Karami and Groves [6].

After a severe drought on Farur Island in two consecutive years 1985–1986, 38 skulls (22 males, 16 females) were collected by B. Farahang Dareshuri. These skulls were studied by M. Karami and five of them (3 males, 2 females) were forwarded to C. P. Groves providing the type material for the subspecies *dareshurii* [6]. Karami and Groves [6] took 23 measurements on each skull of which seven to nine were selected for multivariate analyses. They compared these skulls with other gazelle species, especially with other described subspecies of *G. arabica*, including *G. a. cora* (the common Arabian gazelle, later synonymized with *G. arabica* [14, 15]), “*G. a. erlangeri”* (from the southwestern Arabian Peninsula, probably a pet gazelle and therefore placed in "" in the remaining text, see [16]), and *G. a. muscatensis* (from the Batinah coast of Oman). The results showed that although the Farur gazelles are similar to *G. a. muscatensis* in horn characters (lyrate horns in both sexes, well-formed horns with clear rings in females), they can be differentiated from them and other *G. arabica* taxa, with clearer separation of females than males in discriminant analysis. However, male skulls from Farur were not distinguishable from “*G. a. erlangeri”* in this analysis, despite the fact that they display very different horn shapes (straight horns in “*G. a. erlangeri*”).

Based on these results, Karami and Groves [6] described *G. g. dareshurii* as “a subspecies of *Gazella gazella* (now *G. arabica*, see Bärmann et al. [14]) similar in size and horn shape to *G.[a.] muscatensis* but with longer horns in males; in both sexes the horns are broader across the base, the skull is much narrower, and the nasal bones are posteriorly narrower. Compared to *G. [a.] cora*, it differs additionally in its much smaller size, with shorter horns in the male but longer horns, broader at the base, in the female. Compared to *G. [a.] erlangeri* it differs primarily in its horns, which are outbowed, with the tips turned in, in both sexes. Special comparison with the much larger, straight-horned *G. gazella* from Israel and with the very small, also straight-horned, *G. [a.] farasani* from the Farasan Island, is unnecessary.”

The origin of the Farur gazelles remains enigmatic until today and several hypotheses were put forward on how their presence on the island could be explained: (1) Karami and Groves [6], based on the pers. comm. with M. T. Moinian, suggested that eight individuals of unknown sex had been introduced to Farur Island in 1967 from Kavir National Park (NP) in central Iran. However, no signs of the existence of a possible source population could be found in Kavir NP, which is only inhabited by another gazelle species (*G. bennettii*) [6]. (2) Hemami and Groves [11] suggested that the gazelles from Farur Island might originate from some unspecified place on the Arabian Peninsula. Karami et al. [10] specified this suggestion and mentioned that the gazelles of Farur might be related to *G. a. muscatensis*, an enigmatic subspecies of *G. arabica* inhabiting the coastal plains of north-west Oman. However, both differ in fur coloration with the Farur gazelles having a pale sandy-brown pelage rather than a deep chocolate-brown known from *G. a. muscatensis*. (3) Unconfirmed information from the local people says that Sheikh Oboud Moghuyehie introduced one male and one female to the island in the 1950s from Bandar Moghuyeh (habitat of *G. bennettii*) on the Iranian shores close to Farur (Fig. 1), but no documentation is known corroborating this hypothesis [17]. Thus, the question of the origin of the Farur gazelles remains unclear.

Here, we investigate the phylogenetic relationships and morphological similarity of the Farur gazelles with Arabian mainland *G. arabica* by using molecular and morphometric methods. We hypothesize that (1) *G. a. dareshurii* is a valid subspecies of *G. arabica*, and (2) Farur is the historic habitat that once covered the northern part of the Persian Gulf and this population of mountain gazelles was trapped on the island due to the rising sea level after the LGM.

## Results

### Genetic analyses

Seventeen samples from Farur were successfully sequenced for one or more of the following markers: chromodomain–helicase–DNA-binding protein 2 (CHD2) (669 bp, ten samples), zinc finger protein 618 (ZNF618) (689 bp, nine samples), and cytochrome *b* (cyt *b*) (1140 bp, ten samples). For all three markers, only one haplotype was detected in all samples. Differences in the intron sequences of Farur gazelle compared with *G. arabica* and *G. gazelle* are shown in Table 1.

**Table 1:** Variable sites in the intron sequences of ZNF618 and CHD2

Species	Location	ZNF618	CHD2	Variable sites of ZNF618											Variable sites of CHD2			
				29–31	57	112	136	368	381	403	500	545	558	598	7	305	335	493
Farur gazelle	Iran: Farur Island	OL355296	OL355286	AAG	_	C	G	G	_	T	C	C	T	C	A	C	A	T
*G. gazella*	Palestine: Afik Junction	KU560837	KU560704	_ _ _	_				_		T					Y		W
	Palestine: Yehuda Mountains	KU560838	KU560705	_ _ _	_		R		_		?	?	?	?				
	Palestine: Shomeron	KU560839	KU560706	???	?				_									0
*G. arabica*	Palestine: A'rava Valley	KU560840	KU560707	…	A		A		_									A
	Palestine: A'rava Valley	KU560841	KU560708	…	_		A		_									A
	Saudi Arabia: Farasan Islands	KU560842	KU560709	…	_	T		A	_	C			C	T				
	Oman: Muscat-sur	KU560843	KU560710	…	_				_									W
	KKWRC	KU560844	KU560711	…	_	T		A	G	C		_	C	?	G		C	A

**Fig. 2:**
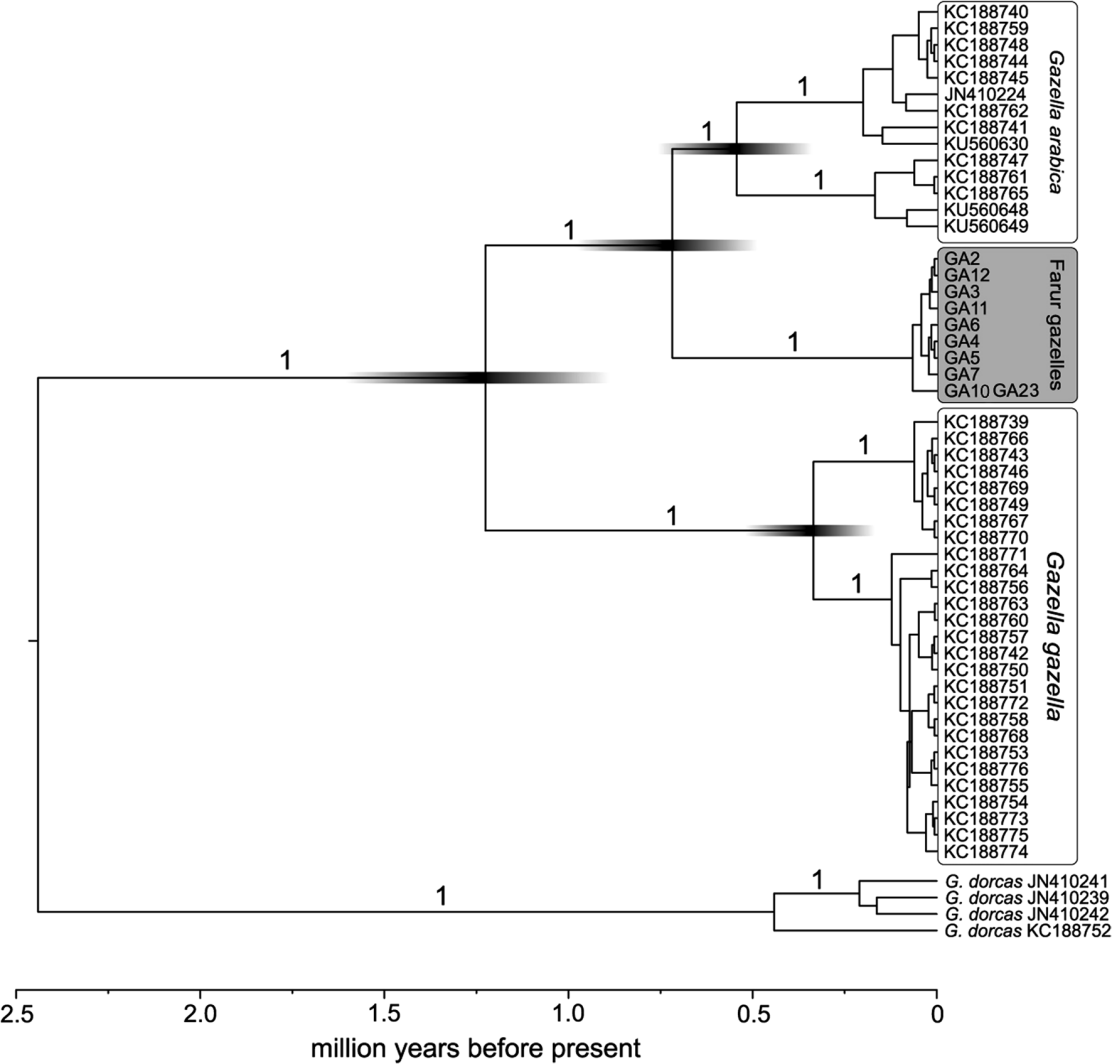
Phylogeny of Mountain gazelles including *G. a. dareshurii* inferred from complete cyt *b* gene sequences using BEAST MC^3^ v.1.7.5 [42]. Numbers above branches are posterior probabilities

**Fig. 3:**
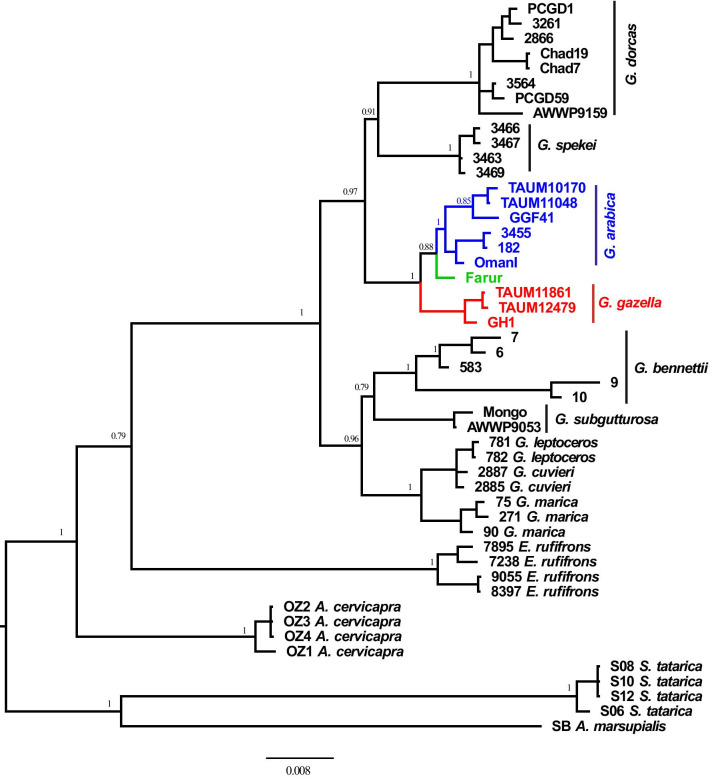
Phylogeny of Mountain gazelles based on the 2521 bp from concatenated analysis of mitochondrial cyt *b* and two nuclear introns (ZNF618 and CHD2). Above branches posterior probability values are reported. The tree was rooted using four related taxa (*Saiga tatarica*, *Antidorcas marsupialis*, *Antilope cervicapra*, and *Eudorcas rufifrons*) as outgroups

The phylogenetic tree of mountain gazelles shows *G. arabica* and *G. gazella* as sister species as expected from previous studies (posterior probability (PP) = 1). Within *G. arabica*, the samples from Farur Island form a monophyletic group (PP = 1) that is placed as sister to all other sequences from all over the mainland of the Arabian Peninsula (Fig. 2 and Fig. 3). According to the molecular clock, the split between the two groups occurred around 0.7 Ma (Fig. 2).

### Morphometric analyses

#### Principal component analyses

**Fig. 4:**
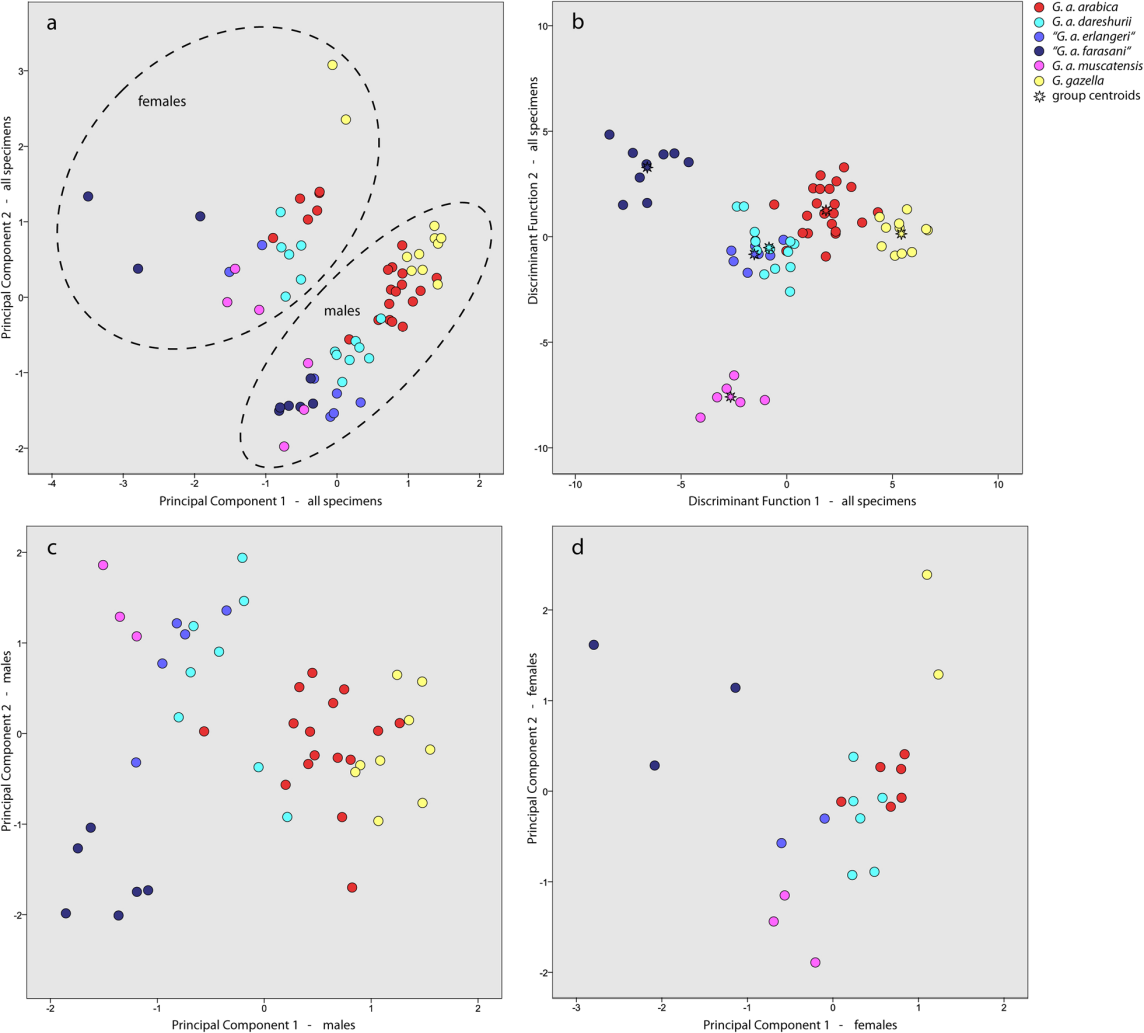
Principal component analysis (**a**, **c**, **d**) and discriminant function analysis (**b**) of gazelle skulls using linear measurements. Analysis of 69 skulls from both sexes using 19 measurements (**a, b**), analysis of 47 male skulls using 19 measurements (**c**), and analysis of 22 female skulls using 15 measurements (**d**). Measurements are described in Table 5

In the first principle component analysis (PCA, Fig. 4a), including all specimens, two main components were found that together account for 74% of the variance of the data set. This analysis clearly separates males and females, so that separate PCAs were also conducted to explore the spread of the data for each sex. However, as only very few female specimens were available, both sexes were analyzed together in the discriminant function analysis (DFA).

In the PCA including only male specimens (Fig. 4c), four principal components were found (explaining 75% of the variance). The first and second components, together accounting for 60% of the variance, distinguish three groups of gazelles with only minimum overlap. The first component, mainly influenced by skull width and length, and occipital height, separates the larger *G. gazella* and *G. a. arabica* from the other groups. The second component, mainly influenced by horn width, horn tip distance, horn length, and braincase height, separates “*G. a. farasani*” from the other groups. Most of the *G. a. dareshurii* males are found in a cluster with “*G. a. erlangeri*” and *G. a. muscatensis*, but two specimens are closer to *G. a. arabica* in morphospace.

For the third PCA, including only females (Fig. 4d), the number of specimens was relatively low. We therefore excluded four measurements that showed very low extraction values (below 0.7) or were only influential in the fourth component in a preliminary analysis, to have a more appropriate parameter-to-specimens ratio. Three main components were found, together describing 86% of the variance of the data. The first component is influenced by measurements describing skull length and width, similarly as in males. But in addition, horn length and diameter, as well as braincase length and height, have a large impact. The second component is mainly influenced by horn distance at the base, maximum horn width, and facial length parameters (DFO, DFH). Together these two components separate the small and short-horned “*G. a. farasani*” as well as *G. a. muscatensis* from the other groups. *G. a. dareshurii* is situated in an intermediate position between *G. a. arabica*, “*G. a. erlangeri*”, and *G. a. muscatensis*.

#### Discriminant function analysis

The DFA including all specimens (Fig. 4b) based on raw measurements had a success rate of 98.6%; only one *G. a. arabica* was misclassified as *G. a. dareshurii*. In cross-validation the success rate was 85.5%. The misclassifications involved almost all *G. arabica* subgroups, including three *G. a. arabicas*, one “*G. a. erlangeri*”*,* and one “*G. a. farasani*” classified as *G. a. dareshurii*, as well as two *G. a. dareshurii* that were classified as *G. a. arabica* and “*G. a. erlangeri*”, respectively (Table 2).

**Table 2:** Results of discriminant function analysis, including cross-validation

Species		Predicted group						Total
		*arabica*	*muscatensis*	*“erlangeri”*	*“farasani”*	*dareshurii*	*G. gazella*	
Original	*arabica*	21 (95.5)	0	0	0	1 (4.5)	0	22
	*muscatensis*	0	6 (100)	0	0	0	0	6
	*“erlangeri”*	0	0	7 (100)	0	0	0	7
	*“farasani”*	0	0	0	9 (100)	0	0	9
	*dareshurii*	0	0	0	0	14 (100)	0	14
	*G. gazella*	0	0	0	0	0	11 (100)	11
Cross-validation	*arabica*	18 (81.8)	0	0	0	3 (13.6)	1 (4.5)	22
	*muscatensis*	0	6 (100)	0	0	0	0	6
	*“erlangeri”*	0	0	6 (85.7)	0	1 (14.3)	0	7
	*“farasani”*	0	0	0	8 (88.9)	1 (11.1)	0	9
	*dareshurii*	2 (14.3)	0	2 (14.3)	0	10 (71.4)	0	14
	*G. gazella*	0	0	0	0	0	11 (100)	11

## Discussion

### Molecular phylogenetic analysis

The phylogenetic analysis places *G. a. dareshurii* with *G. arabica*, as expected from previous analyses based on morphology. However, the Farur specimens are not nested within *G. arabica*, as would be expected if these gazelles were translocated from some Arabian mainland population in historic times, but form the sister-group to all other *G. arabica* (Figs. 2, 3). This implies that this taxon has split from the other Arabian gazelles a long time ago.

### Morphometric analysis

The principal component analyses show that only “*G. a. farasani*” is clearly separate from the other *G. arabica* subgroups. These very small gazelles with very short horns inhabiting the Farasan Islands in the Red Sea were shown to be an ecotype rather than a subspecies of *G. arabica* [18], as high genetic admixture exists between the Farasan and the mainland gazelles (therefore the name is put in “” in this text). The only misclassification that occurred between “*G. a. farasani*” and *G. a. dareshurii* in the cross-validation of the discriminant function analyses involved a female (one female “*G. a. farasani*” classified as *G. a. dareshurii*), which might be caused by the very small numbers of females in our analysis (only three females each for “*G. a. farasani*” and *G. a. muscatensis*, only two females each for “*G. a. erlangeri*” and *G. gazella*). There seems to be no convergent “island morphotype” of Farur and Farasan gazelles that distinguishes them from the mainland gazelles.

A lot more similarity, and misclassification in the DFA, can be observed between *G. a. dareshurii*, “*G. a. erlangeri*”, and *G. a. arabica*. As described by Karami & Groves [6], the Farur gazelles show the closest similarity to “*G. a. erlangeri*” in the PCA. The taxonomic status of “*G. a. erlangeri*” has recently been reviewed [16]. Based on genetic and morphometric analysis, the authors conclude that “*G. a. erlangeri*”, only known from captive populations, is most likely a pet gazelle derived from a darker-coloured variant of *G. arabica*, possibly of the now extinct *G. a. muscatensis*. It is astonishing that the Farur gazelles, despite being isolated on the island for thousands of years, and being restricted to a very small population size (< 1000), have not evolved a distinct morphology. Instead, they mediate between the morphotypes of *G. muscatensis* and “*G. erlangeri*” on the one hand, and *G. arabica* on the other. They demonstrate that the morphospace of *G. arabica* does not display any major gaps between regional subgroups as were found in previous analyses based on available museum specimens.

### The origin of *G. a. dareshurii*

During the LGM, the Persian Gulf was a river valley with a few hilly outcrops along the north-eastern rim [19]. Mountain gazelles might have inhabited these hills, as they are usually found in mountainous regions throughout the Arabian Peninsula [13, 20, 21]. These outlier populations would have been genetically isolated from the Arabian populations for a long time, as the fertile and well hydrated land between them acted as a barrier for dispersal.

After the LGM, about 14,000 BP, the rising sea levels lead to a flooding of the Persian Gulf [2]. During this process, Farur became an island about 10,000 BP [2, 19]. We postulate that the Arabian gazelles on Farur survived on the island since that time, despite being restricted to an extremely small population size. In historical times, only 300–500 gazelles inhabited the island at any one time. Possibly other outlier populations could have existed on other islands or in the mountain areas on the Iranian coast, but our extensive efforts to collect samples from all possible habitats did not reveal any other populations of *G. arabica* in Iran. So we can only speculate on why they did not survive. On the mainland and the larger islands, competition with other gazelle species, especially *G. bennettii* which is also adapted to desert conditions, might have led to the local extinction of *G. arabica*. On the smaller islands, the catastrophic effects of drought and flooding could have been too severe for the long-term survival of a small and isolated population of gazelles. On Farur, these natural threats did not lead to extinction, but now anthropogenic threats might negatively affect the Farur gazelles.

### Threats for the Farur gazelles

#### Natural threats

Although people were living on Farur in the past (< 1950), nowadays neither humans nor carnivores inhabit the island. Thus, the gazelles living on the island have no natural predators. Diseases and droughts were suggested to be the major threats to their survival [11]. Farur is under the influence of Indian summer (the middle of June–the middle of September) and winter (December–March) monsoon. Without doubt, the main natural threat is intermittent drought which was the main reason for the population decline in 1986 (low precipitation during the winter monsoon). In several years, also flooding events caused by heavy rain during monsoon were responsible for population declines where carcasses of gazelles were found in the valleys (Saman Ghasemi and Meisam Ghasemi, personal observations). However, diseases were so far not recorded to occur in this gazelle population, probably due to the geographic isolation from other wild or captive bovids. But diseases could potentially have a large impact, as they most likely would infect the entire population. Therefore, intermittent drought (leading to food limitation) and flooding are possibly two main natural threats that might act as balancing factors in the absence of natural predators. They naturally restrict population growths and therewith prevent severe overgrazing of the island which otherwise could lead to a collapse of the gazelle population.

#### Inbreeding

It seems surprising that such a small population of gazelles that persisted on a tiny island without genetic exchange for thousands of years has not suffered from, or even gone extinct by inbreeding. However, it is not the amount of genetic diversity, but the absence of large amounts of strongly deleterious mutations that is responsible for the viability of small populations [22, 23]. A genomic study on wild foxes inhabiting the Channel Islands in California for more than 9000 years has revealed that these populations show an extremely low amount of moderately to strongly deleterious mutations compared to mainland foxes [24]. This can be explained by genomic purging, i.e., the wiping out of moderately and strongly deleterious recessive mutations due to increased selection pressure, as these mutations are more often found in homozygosity in small populations [25]. We think that the situation on Farur is very similar, so that the absence of inbreeding depression can be explained by the very long isolation and the consistently small population, even before Farur became an island. The accumulation of mildly deleterious mutations that likely occurs in such small populations, as was demonstrated for Alpine ibex [26], still leads to a reduction of the fitness, but in an environment without predators this seems to have a low impact. In any case, genomic studies of these gazelles would be highly desirable to gain more information on genomic purging in island populations.

#### Anthropogenic threats

Farur gazelles adapted to the natural situation of the island, so every anthropogenic intervention can be a threat to the survival of the gazelles. Umbrella thorn (*Acacia tortilis*) is the main food source during the dry season, but a newly introduced tree, mesquite (*Prosopis juliflora*)*,* now invasively occupies some part of the island [27]. This alien species is a potential threat for the *Acacia* and therefore also for the gazelles.

Recently, the Iranian navy has set up a camp on the island, increasing gazelle-human contact: in the dry season, gazelles tend to come close to the settlements in search of food and water. Several water reservoirs were built on the island to reduce the effects of drought for gazelles. Although the presence of the naval forces led to a complete stop of illegal hunting activities, the construction activities commissioned by the navy affect the natural habitat of gazelles. Iranian Department of Environment (DoE) authorized hunting for the first and last time in 2010 when wrongly considering the Farur gazelles to belong to *G. bennettii*, a common gazelle of the Iranian mainland.

## Conclusion and implications for conservation

Farur gazelles are not only a subspecies of *G. arabica*, but also are a remarkable relict population estimated to have split from other *G. arabica* populations 0.7 Ma and survived on Farur in isolation (10,000 BP). Being trapped on the small island led to adaptation to the island’s nature with drought and flood acting as balancing forces to regulate population growth in the absence of natural predators. Conservation actions are necessary for this relict population as its long-lasting separate evolutionary history might have led to the acquisition of genomic changes in adaptation to the specific island’s requirements. It could therefore serve as an example of local adaptation [28], be used as a model for the assessment of evolutionary change and genomic purging [24, 29], and represent a case study for biogeographical studies [30]. Farur gazelles are the only recorded population of *G. a. dareshurii*, so they should be treated as a separate conservation and management unit [31]. Therefore, conservation actions should aim at ensuring the survival of the population within its natural environment, and possibly introducing the species to other islands (not inhabited by gazelles) or the Iranian mainland (strictly isolated from other gazelle populations) when the Farur population increases to more than the island’s carrying capacity.

## Methods

### Farur Island

Farur Island is located in the northern middle part of the Persian Gulf (Fig. 1). The shortest distance to the mainland is around 22 km, with Boustaneh as the nearest point on the Iranian mainland. The greatest length and width are 7.5 and 4.5 km respectively, and the area is 28.48 square kilometers with an elevation ranging from 0 to 140 m above sea level. The island is located between Kish Island in the west, Qeshm, Greater Tunb, and Lesser Tunb Islands in the east, and Siri and Abu Musa Islands in the south and southeast (Fig. 1). Farur Island is a protected area under the DoE since 1979. It is the only island in the Persian Gulf inhabited by Arabian mountain gazelle.

The island surface is uneven and hilly with several peaks on its central and western parts. The highest peak is 145 m and is located in the west of the island. Most of the island area is at an altitude of 50 m. Grass, shrub, and mostly trees that have adapted to the hot weather cover the island. Umbrella thorn (*A. tortilis*) is distributed on the whole island with the highest density in the valleys. Mesquite (*P. juliflora*) is an invasive species that recently arrived on the island [27].

The climate is tropical with seasonal mean temperatures of 27 °C in spring (March–May), 34 °C in summer (June–August), 29 °C in autumn (September–November), and 20 °C in winter (December–February) based on the Bandar Lengeh weather station (1966–2017) as the nearest synoptic weather station to the island. Annual precipitation is 133 mm and relative humidity is high during the year with over 90% on some days. The Persian Gulf is under the influence of Indian summer monsoon in the boreal summer from the middle of June until the middle of September, and Indian winter monsoon in the boreal winter between December and March with weaker, dry, and cold northeasterly winds compared to the strong southwestern monsoonal winds [32, 33]. Total monthly precipitation (1966–2017) shows that December (28.20), January (34.05), February (26.52), and March (26.77) are raining months, and after April (6.17), the total monthly precipitation is less than one millimeter in May, June, and July. It seems that August (2.02) and September (1.02) are monsoon-influenced months in the summer, and October (0.25) and November (6.86) are the months after disappearing Indian summer monsoon.

#### Farur gazelles

**Fig. 5:**
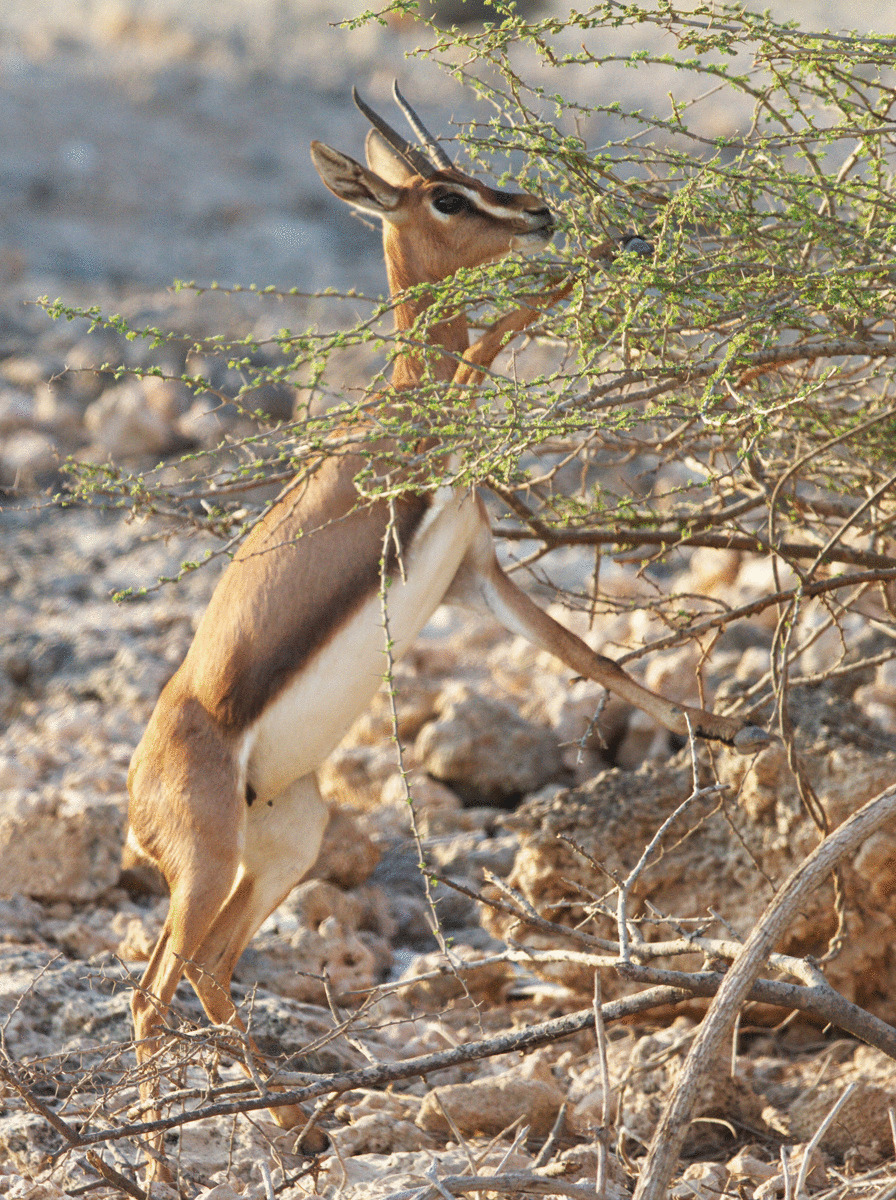
*G. a. dareshurii* on Farur Island browsing on umbrella thorn (*A. tortilis*) tree leaves. Photo by Meisam Ghasemi

Based on the observation of the DoE, the breeding season of the Farur gazelles starts in November before the beginning of boreal winter (December–March), when monthly precipitation increases (average total monthly precipitation: 28.88) and temperatures decrease (average total monthly temperature: 20.77), probably the best time for young to be born. Around 350 (range: 187–519) gazelle individuals exist on Farur at any one time. They are mainly browsers (diurnal and partially nocturnal), feeding on foliage, flowers, and seed pods of *A. tortilis* (Fig. 5) and other shrubs, but also graze on grasses and herbs, like other populations of *G. gazella* and *G. arabica* do [34–36]. *Acacia* trees produce a large number of pods that are eaten by gazelles. In the dry season the gazelles recently started to feed on forage provided by the DoE. The soil of the island is bare on many days of the year, and it seems that the percentage of grass cover and the height of *A. tortilis* are important environmental variables affecting the presence of *G. a dareshurii* in spring [37].

### DNA extraction, amplification, and sequencing

Thirty tissue samples were collected from dead gazelles on the island after a flood event in 2011 (Table 3) and preserved in 96% ethanol in sterile 15 ml tubes.

**Table 3:** List of tissue samples collected from Farur Island

Museum ID	ID	Location	Cyt *b*		CR	Nuclear introns		Sex
			Partial	Complete		CHD2	ZNF618	
G- gda 25006	GA19	Iran: Farur Island	KF420269		KF712336			Male
G- gda 25007	GA20	Iran: Farur Island	KF420270		KF712337			Male
G- gda 25008	GA21	Iran: Farur Island	KF420271					Male
G- gda 25021	GA1	Iran: Farur Island	KF420272		KF712338			Female
G- gda 25022	GA2	Iran: Farur Island		OL347679		OL355286	OL355296	Female
G- gda 25023	GA3	Iran: Farur Island		OL347680		OL355287	OL355297	Male
G- gda 25024	GA4	Iran: Farur Island	KF420273	OL347681		OL355288	OL355298	Male
G- gda 25025	GA5	Iran: Farur Island		OL347682				
G- gda 25026	GA6	Iran: Farur Island	KF420274	OL347683	KF712339			
G- gda 25027	GA7	Iran: Farur Island	KF420275	OL347684				
G- gda 25028	GA8	Iran: Farur Island	KF420276		KF712340			
G- gda 25029	GA9	Iran: Farur Island	KF420277					
G- gda 25030	GA10	Iran: Farur Island		OL347685				Female
G- gda 25031	GA11	Iran: Farur Island		OL347686				Male
G- gda 25032	GA12	Iran: Farur Island		OL347687				Female
G- gda 25040	GA16	Iran: Farur Island				OL355289	OL355299	Male
G- gda 25047	GA18	Iran: Farur Island	KF420278					Female
G be-25265	GA23	Iran: Farur Island		OL347688				
	GA24	Iran: Farur Island				OL355290	OL355300	
	GA26	Iran: Farur Island				OL355291	-	
	GA27	Iran: Farur Island				OL355292	OL355301	
	GA28	Iran: Farur Island				OL355293	OL355302	
	GA29	Iran: Farur Island				OL355294	OL355303	
	GA30	Iran: Farur Island				OL355295	OL355304	
GH1		Palestine: Afik Junction		KU560629		KU560704	KU560837	
TAUM11861		Palestine: Yehuda Mountains		KC188775		KU560705	KU560838	
TAUM12479		Palestine: Shomeron		KC188774		KU560706	KU560839	
TAUM10170		Palestine: A'rava Valley		KC188740		KU560707	KU560840	
TAUM11048		Palestine: A'rava Valley		KC188759		KU560708	KU560841	
GGF41		Saudi Arabia: Farasan Islands		KU560630		KU560709	KU560842	
OmanI		Oman: Muscat-sur		KU560648		KU560710	KU560843	
3455		Captive animal held at King Khalid Wildlife Research Center		KU560649		KU560711	KU560844	

DNA was extracted using phenol–chloroform methods [38]. For amplification of the complete coding region of the mitochondrial cyt *b* we used the primers L14724 and H15915 [39]. The reaction mixture was prepared in 25 μl volume, containing 1 unit of Euro Taq DNA polymerase, 10 µM Tris–HCl, 30 µM KCl, 1.5 mM MgCl_2_, 250 µM of each dNTP and 2 pmol primers (Bioneer, South Korea). The thermocycling was performed as follows: initial denaturation (180 s at 95 °C), followed by five cycle steps of 60 s at 94 °C (denaturation), 90 s at 45 °C (primer annealing) and 90 s at 72 °C (elongation), then 40 cycle steps of 60 s at 94 °C, 60 s at 50 °C and 90 s at 72 °C, and lastly, a final extension step (600 s at 72 °C) [39]. Double-strand cycle Sanger sequencing was performed using the Big Dye Terminator Cycle Sequencing kit v.3.1 (Applied BioSystems) and electrophoresis of the purified sequencing product was carried out on an ABI PRISM 3730xl automatic sequencer.

For a phylogenetic analysis of the genus *Gazella*, Lerp et al. [40, 41] published a new set of nuclear intron markers. Two of the six markers (CHD2 and ZNF618) were selected for the amplification using the primers from Lerp et al. [41] as they showed a good distinction between *G. gazella* and *G. arabica*.

The PCR was carried out in a GeneAmp 2720 Thermo Cycler (Applied Biosystems) using QIAGEN Multiplex PCR Kit in 20 μl volume, containing 2 µl Q-Solution, 10 µl QIAGEN Multiplex PCR Master Mix (including HotStarTaq DNA Polymerase, QIAGEN Multiplex PCR Buffer, and dNTP Mix), and 1.6 µl of each primer (10 pmol/µl) using the following protocol: 15 min at 95 °C (initial step), followed by 38 cycles of 35 s at 95 °C, 60 s at 60 °C, and 60 s at 72 °C, and finally 10 min at 72 °C (final elongation). PCR products were purified using 6 µl of HT ExoSAP-IT (Thermo Scientific). Purified PCR products were sent off to Macrogen for Sanger Sequencing. Cyt *b* and two nuclear introns sequences were edited for correction with SeqScape v.2.6 (Applied Biosystems). New sequences were submitted to GenBank (cyt *b*: OL347679-OL347688, CHD2: OL355286-OL355295, ZNF618: OL355296-OL355304, Table 3).

### Phylogenetic analyses

#### Cyt *b*

A mitochondrial cyt *b* sequence alignment was constructed including ten new sequences from Farur gazelles (Acc. No: OL347679-OL347688) and 45 sequences already published in GenBank (Acc. No.: see Fig. 2) covering the two species of mountain gazelles *Gazella arabica* and *G. gazella*, and *G. dorcas* (as closest relative to the ingroup for rooting the tree). This alignment was used for the cyt *b* phylogenetic reconstruction. A Bayesian analysis was performed in BEAST MC^3^ v.1.7.5 [42]. jModelTest v.2.1.1 [43] identified HKY + Γ as the best fitting substitution model. We used molecular clock data estimates inferred for *G. dorcas* [39] and ran MC^3^ simulations with 10^7^ generations, discarding the first 10% of the runs as burn-in.

#### Concatenated analysis of cyt *b* and two nuclear introns

Based on the alignment by Lerp et al. [41] we created a concatenated alignment of all three markers, i.e., cyt *b* and two nuclear introns (ZNF618 and CHD2), adding for each marker the single haplotype of the Farur gazelles to the sequences provided by Lerp et al. [41]. Sequences were aligned using the Clustal W algorithm [44] implemented in Mega v.5 [45], and final adjustments were made by eye. The final alignment has 2521 bp.

The best-fitting partitioning scheme and nucleotide substitution models were estimated using greedy search algorithm with PhyML [46] in PartitionFinder v.2.1.1 [47, 48]. We tested among partitioning schemes including division of protein-coding genes of cyt *b* into 1st, 2nd, and 3rd codon positions and two nuclear intron partitions. Models were selected by the Bayesian information criterion (BIC). We found the optimal partitioning scheme includes four partitions (optimal models are indicated in brackets) 1st codon of cyt *b* (K80 + I), 2nd codon of cyt *b* and CHD2 (HKY + I), 3rd codon of cyt *b* (HKY + I), and 4th ZNF618 (HKY + Γ). Bayesian inference analyses were carried out in MrBayes v.3.2 [49] with two independent runs of four Markov chains (one cold and three heated) over 10,000,000 generations and sampling every 1000 generations. The first 25% of the sampled trees and estimated parameters were discarded as burn-in. Convergence of the model parameters was monitored using the program Tracer v.1.7.1 [50]. The consensus phylogenetic tree was then edited in FigTree v.1.4.4 (http://tree.bio.ed.ac.uk/software/figtree/).

### Morphometric analysis

In the present study, 14 skulls of gazelles from Farur Island (8 males, 6 females) were measured, including 13 skulls from the Museum of Hormozgan Department of Environment (HDoE) and one from the Isfahan University of Technology (IUT). The 13 skulls from HDoE were collected during fieldwork by HDoE in 2011. The skull in IUT belongs to the type series that was collected after a drought in 1986 by B. Farahang Dareshuri.

Up to 50 measurements per skull were taken by D.F. based on the method described in Bärmann et al. [14]. This data set is complemented with data from Bärmann et al. [14] and Wronski et al. [16], including 22 *G. a. arabica* (16 males, 6 females), 7 “*G. a. erlangeri*” (5 males, 2 females), 6 *G. a. muscatensis* (3 males, 3 females), 9 “*G. a. farasani*” (6 males, 3 females), and 11 *G. gazella* (9 males, 1 female) specimens (Table 4). Missing measurements due to incomplete skulls were replaced with average values of the other specimens belonging to the same taxon and sex. Nineteen measurements (Table 5) were included in the final analyses. All values were log10-transformed as recommended by Keene [51]. The data were explored using PCA and DFA, with cross-validation to test for the distinctness of *G. a. dareshurii* from other *G. arabica* subspecies (Fig. 4, Table 2). All morphometric analyses were conducted with SPSS v.24.

**Table 4:** Gazelle skulls included in the morphometric analyses

Taxon	Collection	Accession No.	Sex
*G. a. arabica*	KKWRC	G1047	Male
*G. a. arabica*	KKWRC	G1095	Male
*G. a. arabica*	KKWRC	G1117	Male
*G. a. arabica*	KKWRC	G1173	Male
*G. a. arabica*	KKWRC	G1183	Male
*G. a. arabica*	KKWRC	G1189	Male
*G. a. arabica*	KKWRC	G1192	Male
*G. a. arabica*	KKWRC	G1334	Male
*G. a. arabica*	KKWRC	G1478	Male
*G. a. arabica*	KKWRC	G1541	Male
*G. a. arabica*	KKWRC	G1551	Male
*G. a. arabica*	KKWRC	G1583	Male
*G. a. arabica*	KKWRC	G1593	Male
*G. a. arabica*	KKWRC	G1613	Male
*G. a. arabica*	KKWRC	G1637	Male
*G. a. arabica*	KKWRC	G711	Male
*G. a. arabica*	KKWRC	G1176	Female
*G. a. arabica*	KKWRC	G1208	Female
*G. a. arabica*	KKWRC	G1540	Female
*G. a. arabica*	KKWRC	G1740	Female
*G. a. arabica*	KKWRC	G584	Female
*G. a. arabica*	KKWRC	G642	Female
*G. a. muscatensis*	HI	HZM 11.4114	Male
*G. a. muscatensis*	HI	HZM 26.4534	Male
*G. a. muscatensis*	HI	HZM 6.4049	Male
*G. a. muscatensis*	HI	HZM 12.4115	Female
*G. a. muscatensis*	HI	HZM 4.4047	Female
*G. a. muscatensis*	HI	HZM 7.4050	Female
*“G. a. erlangeri”*	KKWRC	M168	Male
*“G. a. erlangeri”*	KKWRC	M187	Male
*“G. a. erlangeri”*	KKWRC	M208	Male
*“G. a. erlangeri”*	KKWRC	M51	Male
*“G. a. erlangeri”*	KKWRC	M91	Male
*“G. a. erlangeri”*	KKWRC	M117	Female
*“G. a. erlangeri”*	KKWRC	M02	Female
*“G. a. farasani”*	KKWRC	F_22	Male
*“G. a. farasani”*	KKWRC	F_100	Male
*“G. a. farasani”*	KKWRC	F_8	Male
*“G. a. farasani”*	KKWRC	F_13	Male
*“G. a. farasani”*	KKWRC	F_38	Male
*“G. a. farasani”*	KKWRC	F_5	Male
*“G. a. farasani”*	KKWRC	F_28	Female
*“G. a. farasani”*	KKWRC	F_2	Female
*“G. a. farasani”*	KKWRC	F_7	Female
*G. a. dareshurii*	HDoE	14GADS	Male
*G. a. dareshurii*	HDoE	12GADS	Male
*G. a. dareshurii*	HDoE	6GADS	Male
*G. a. dareshurii*	HDoE	5GADS	Male
*G. a. dareshurii*	HDoE	4GADS	Male
*G. a. dareshurii*	HDoE	3GADS	Male
*G. a. dareshurii*	HDoE	2GADS	Male
*G. a. dareshurii*	IUT	1GADS	Male
*G. a. dareshurii*	HDoE	13GADS	Female
*G. a. dareshurii*	HDoE	9GADS	Female
*G. a. dareshurii*	HDoE	8GADS	Female
*G. a. dareshurii*	HDoE	7GADS	Female
*G. a. dareshurii*	HDoE	11GADS	Female
*G. a. dareshurii*	HDoE	10GADS	Female
*G. gazella*	BMNH	10.3.12.16	Male
*G. gazella*	BMNH	10.3.12.17	Male
*G. gazella*	MfN	ZMB_17683	Male
*G. gazella*	BMNH	4.12.18.1	Male
*G. gazella*	MfN	ZMB_58699	Male
*G. gazella*	MfN	ZMB_58814	Male
*G. gazella*	MfN	ZMB_58815	Male
*G. gazella*	MfN	ZMB_58421	Male
*G. gazella*	MfN	ZMB_58813	Male
*G. gazella*	BMNH	4.12.16.2	Female
*G. gazella*	MfN	ZMB_58418	Female

Collections: British Museum of Natural History London, UK (BMNH), Museum of Hormozgan Department of Environment, Iran (HDoE), Museum of the Harrison Institute in Sevenoaks, UK (HI), Isfahan University of Technology, Iran (IUT), King Khalid Wildlife Research Center, Saudi Arabia (KKWRC), Museum für Naturkunde Berlin, Germany (MfN)

**Table 5:** Skull measurements used in the morphometric analyses

	Factor loadings in each component		Extraction communalities	Description
	C1	C2		
DFH	0.696	*0.561*	0.800	Distance front to horns
DFO	*0.825*	0.259	0.748	Distance front to orbit
DH	− 0.485	*0.667*	0.680	Distance between horns pedicles
DOC	0.677	− 0.104	0.470	Distance orbit to condyle (measured parallel to tooth row)
HD1	*0.853*	− 0.434	0.916	Horn pedicle diameter 1 (medio-lateral)
HD2	*0.840*	− 0.500	0.955	Horn pedicle diameter 2 (antero-posterior)
HL1 r	*0.800*	− 0.424	0.820	Horn length, distance between the base of the horn sheath and the horn tip
HTD	0.656	*− 0.551*	0.734	Horn tip distance
IB	0.721	0.194	0.558	Inter-bullae distance
LF + P1	*0.901*	0.167	0.841	Length of frontal + parietal
LL	0.767	0.199	0.629	Length of lacrimal (maximum length of facial part)
LP	0.668	0.396	0.602	Length of parietal
MWH	0.692	− *0.600*	0.839	Maximum width of horns sheaths
OD	0.676	0.403	0.619	Orbit diameter (parallel to tooth row)
OHB	*0.846*	− 0.010	0.717	Occipital height, braincase complete
OHO	*0.901*	0.029	0.812	Occipital height, occiput only (dorsal of foramen magnum)
WAO	*0.888*	0.271	0.862	Width across orbits (maximum width of frontals)
WB	0.781	0.367	0.745	Width of braincase
WPP	0.839	0.204	0.745	Width across paroccipital processes
Eigenvalues	11.297	2.794	0.748	
% of Variance	59.456	14.707	0.680	

Measurements with highest extraction communalities for the respective component are in italics

## Data Availability

DNA sequences have been deposited in GenBank under the accession no: OL347679-OL347688 and OL355286-OL355304.
